# Polyhydroxy Molecular Regulator Enabled Interfacial Microenvironment Programming for Alkaline Zinc–Iron Flow Batteries

**DOI:** 10.1007/s40820-026-02333-2

**Published:** 2026-09-14

**Authors:** Hui Chen, Lukang Han, Shaohua Han, Weijie Fan, Xuhong Yin, Junqing Pan, Manal S. Ebaid, Yuxi Song, Fuyu Chen, Jiang Zhou

**Affiliations:** 1 School of Materials Science and Engineering, Yancheng Institute of Technology, Yancheng, 224051 People’s Republic of China; 2 School of Materials Science and Engineering, Hunan Provincial Key Laboratory of Electronic Packaging and Advanced Functional Materials, Central South University, Changsha, 410083 People’s Republic of China; 3 State Key Laboratory of Chemical Resource Engineering, College of Chemistry, Beijing University of Chemical Technology, Beijing, 100029 People’s Republic of China; 4 Department of Chemistry, College of Science, Northern Border University, Arar, 73213 Saudi Arabia

**Keywords:** Hydrogen-bond network, Interfacial microenvironment programming, Zincate transport, Polyhydroxy molecular regulator, Zinc–iron flow batteries

## Abstract

Alkaline zinc–iron flow batteries (ZIFBs) are attractive for large-scale energy storage because of their low cost, intrinsic safety, and scalable architecture, yet their practical deployment remains hindered by hydrogen evolution and uncontrolled Zn deposition in three-dimensional porous electrodes. Here, we report that isomalt acts as a polyhydroxy molecular regulator that reconfigures the interfacial microenvironment of porous carbon felt, thereby endowing the porous electrode with a more deposition-directing function for reversible Zn plating/stripping. In the bulk electrolyte, isomalt reconstructs the local hydrogen-bond network, lowers water activity, and suppresses hydrogen evolution. At the carbon/electrolyte interface, its preferential lateral adsorption establishes a hydroxyl-rich interphase that homogenizes zincate transport and directs Zn growth toward a preferred (002) orientation, thus promoting compact and reversible deposition. This interfacial microenvironment programming markedly reduces the charge-transfer resistance and activation barrier of the Zn anode, enabling stable cycling for over 500 cycles at 20 mA cm^−2^. In full-cell tests, the optimized ZIFB delivers a high energy efficiency of 87.5%, substantially outperforming the pristine electrolyte. Beyond identifying a low-cost green additive, this work establishes polyhydroxy molecules as interfacial programming units for three-dimensional porous electrodes, offering a new design concept for directional metal deposition in aqueous flow-battery systems.

**Supplementary Information:**

The online version contains supplementary material available at https://doi.org/10.1007/s40820-026-02333-2.

## Highlights

- Isomalt acts as a polyhydroxy molecular regulator that couples bulk-electrolyte reconstruction with interfacial microenvironment programming.
- Isomalt reorganizes the hydrogen-bond network and establishes a hydroxyl-rich interphase through preferential lateral adsorption on carbon.
- This coupled regulation enables more reversible Zn plating/stripping and durable alkaline Zn-Fe flow batteries with high efficiency and long cycling stability.

**Supplementary Information:**

The online version contains supplementary material available at https://doi.org/10.1007/s40820-026-02333-2.

## Graphical abstract

**Figure:**
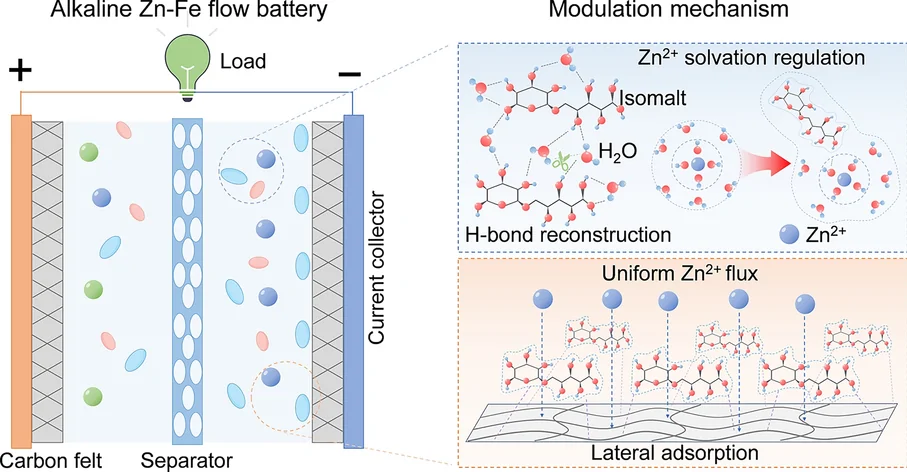

## Introduction

Redox flow batteries (RFBs) are promising for grid-scale energy storage because of their decoupled power and energy output, high intrinsic safety, and scalable architecture [1–3]. Among them, zinc-based systems are particularly attractive owing to their competitive energy density, low cost, and abundant zinc resources [4–6]. Within this family, zinc–iron flow batteries (ZIFBs) have received increasing attention because they combine favorable electrochemical features with clear economic advantages [7–9]. However, their practical deployment remains limited by the instability of the Zn anode during repeated plating/stripping [10–12]. In porous electrodes, uncontrolled Zn deposition readily induces dendritic growth, internal short-circuit risk, and rapid capacity decay [13, 14], while parasitic hydrogen evolution further undermines Zn reversibility, lowers coulombic efficiency, and accelerates electrode degradation [15–17]. Considerable efforts have therefore focused on membrane modification, electrolyte design, and electrode surface engineering [18–21]. Among these strategies, electrolyte regulation is particularly attractive, as it enables system-level control of Zn deposition behavior without significantly increasing device complexity or cost.

Recent advances in electrolyte design have mainly followed three interconnected directions: reconstructing the coordination environment of Zn species, regulating hydrogen-bond (H-bond) networks, and stabilizing the electrode/electrolyte interface to promote reversible plating/stripping [22–25]. Electron-donating ligands can partially replace water in the coordination structure and suppress parasitic reactions [26, 27], but their limited interfacial affinity and added decoordination penalty often constrain deposition kinetics [28–30]. H-bond regulation through multidentate chelators or functional acids can reorganize water structure and reduce proton activity near the interface [31–33], yet excessive intermolecular association may increase viscosity and hinder ion transport. Additive-derived adsorption layers can also homogenize ion flux and improve deposition morphology [34–36]. However, their protective efficacy is often concentration-sensitive and prone to degradation under high-rate or long-term cycling due to additive consumption or uneven coverage.

Nevertheless, these approaches are still largely framed as bulk-electrolyte optimization or static interfacial passivation and seldom address a more fundamental issue in flow batteries: the local chemical and transport environment inside three-dimensional porous carbon electrodes remains largely unprogrammed, even though it directly governs where and how Zn deposition occurs. This challenge suggests that stabilizing Zn anodes in flow batteries requires more than suppressing side reactions or smoothing deposition; it calls for redefining the role of the porous electrode itself. Rather than treating carbon felt as a passive current collector, it may be possible to endow it with interfacial functionality through molecularly programmed adsorption, so that the local microenvironment within the porous framework can actively direct Zn deposition. Polyhydroxy compounds are attractive for this purpose because their abundant hydroxyl groups can simultaneously interact with water and carbon surfaces, enabling coupled regulation of H-bond organization in the bulk electrolyte and adsorption-mediated interfacial structuring on carbon [37–40]. Among them, isomalt, a naturally derived sugar alcohol with a sterically rigid backbone and densely distributed hydroxyl groups, is particularly promising. As depicted in Table S1, its molecular structure is well suited to both electrolyte reconstruction and interfacial organization, compared with previously reported common additives.

**Fig. 1:**
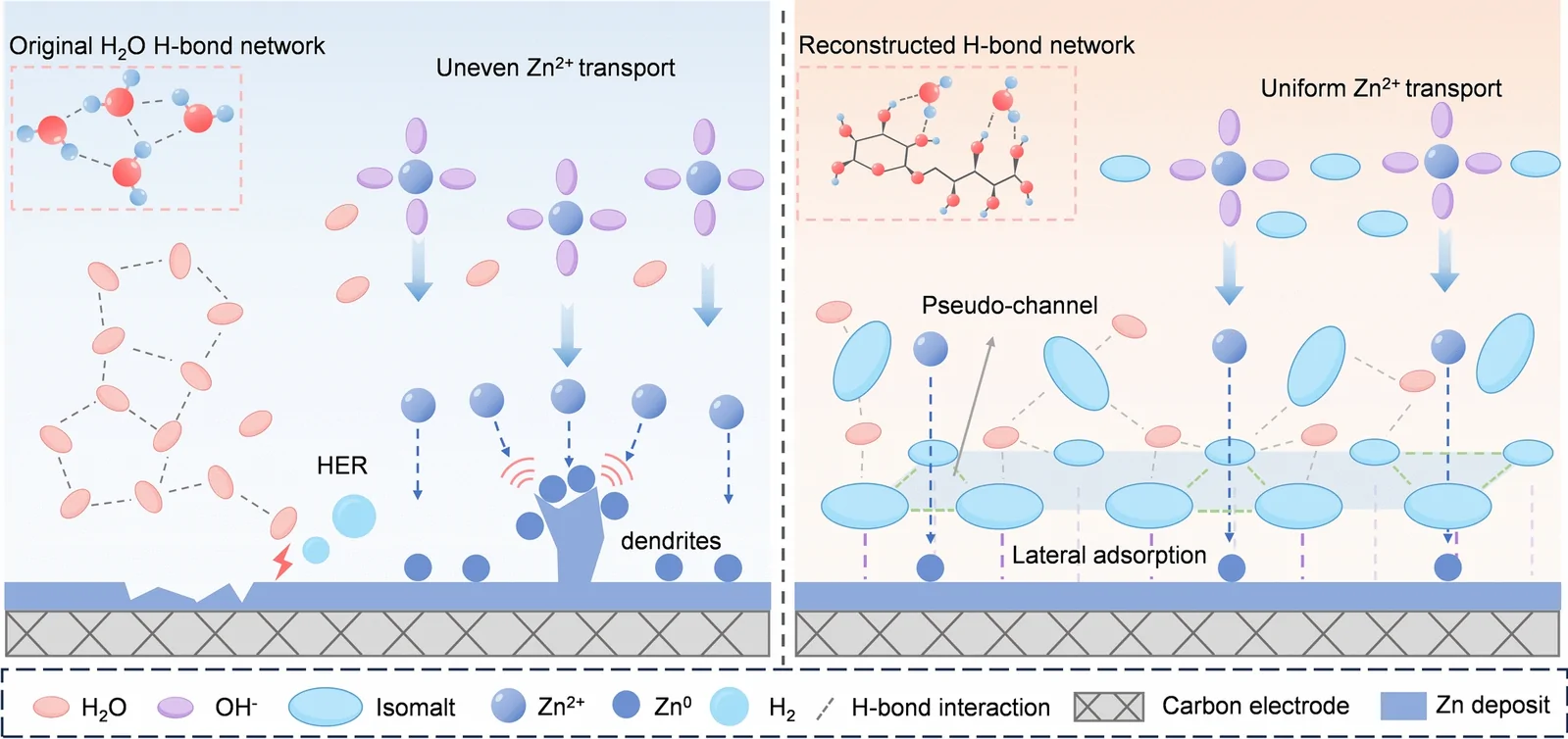
Mechanism of multifunctional regulation by isomalt

Herein, we report isomalt as a polyhydroxy molecular regulator for alkaline ZIFBs and show that its role extends beyond that of a conventional electrolyte additive. As illustrated in Fig. 1, isomalt reconstructs the local H-bond network in the electrolyte, lowers water activity, and suppresses parasitic hydrogen evolution. More importantly, its preferential lateral adsorption on carbon establishes a hydroxyl-rich interfacial microenvironment on porous carbon felt, which homogenizes local zincate transport and directs Zn deposition toward a more favorable growth mode. Through this process, the porous carbon electrode is no longer merely a passive current collector, but instead becomes a deposition-directing substrate for reversible Zn plating/stripping. This coupled regulation of the bulk electrolyte and porous-electrode interface simultaneously suppresses side reactions, improves deposition uniformity, and mitigates dendritic growth during extended cycling. Combined with theoretical calculations and interfacial adsorption analysis, our results identify polyhydroxy-molecule-enabled interfacial microenvironment programming as a new strategy for stabilizing Zn anodes in aqueous flow-battery systems and provide theoretical support for the screening and application of additives with analogous structures and functionalities.

## Experimental Section

### Materials

All reagents used in this study were of analytical grade and employed without further purification. Isomalt (purity 99%) was purchased from Macklin Biochemical Co., Ltd. (Shanghai, China). Zinc oxide (ZnO), potassium hydroxide (KOH), and sodium ferrocyanide (Na_4_[Fe(CN)_6_]) were obtained from commercial suppliers and used as received. Carbon felt (CF) was employed as the electrode substrate after pretreatment by acid washing and drying to remove surface impurities and enhance electrochemical activity, while the zinc foil was rinsed with 0.5 M HCl (Xinyue Chemical Reagent, China) to remove the contaminants and then cleaned up with deionized water.

### Electrolyte Composition

The alkaline anolyte was prepared by dissolving 0.2 M ZnO and 3.4 M KOH in deionized water under constant stirring at 40 °C until a clear solution was obtained (pristine electrolyte). Isomalt was introduced into the base electrolyte at varying concentrations (0, 0.5, 1, 2, and 3 wt%) to investigate its effect as a functional additive. The catholyte was composed of 0.4 M Na_4_[Fe(CN)_6_] and 3 M KOH. All electrolytes were filtered to remove insoluble impurities and stored under a nitrogen atmosphere to minimize oxidative degradation and ensure chemical stability before use.

### Characterization

The morphology was analyzed using scanning electron microscopy (SEM, FEI Nova Nano SEM 450), in conjunction with energy-dispersive X-ray spectroscopy (EDS, Oxford AZtec X-MaxN 80). Surface topography was imaged by atomic force microscopy (AFM, Picoplus 2500, USA). X-ray diffraction (XRD, PANalytical X’Pert^3^ Powder, Cu Kα, *λ* = 1.5406 Å) radiation was conducted ranging from 30° to 90°. X-ray photoelectron spectroscopy (XPS, Thermo ESCALAB 250Xi, 10 kV) was used to probe the surface chemical composition and the oxidation states of the elements on the zinc-deposited CF electrode. Fourier transform infrared spectroscopy (FTIR, Thermo Nicolet NEXUS-670) was conducted to investigate the chemical interactions. The Raman spectra (Horiba France SAS, LabRAM Odyssey) were collected using a 532-nm excitation laser with a laser power of 10 mW. Ultraviolet–visible spectroscopy (UV–Vis, Shimadzu UV-2450) was employed to assess the optical absorbance behavior of electrolytes with varying isomalt concentrations. The viscosity of electrolyte was tested using (DV2T, AMETEK Brookfield, USA). The porosity of electrodes was investigated by mercury intrusion porosimetry (MIP) using a Micromeritics AutoPore V 9600 apparatus (version 2.03.00, Micromeritics Instrument Corporation, USA). The analysis was performed in a pressure range from 0.10 psia to 61,000.00 psia. The contact angle of mercury was set to 130°, and the mercury temperature was maintained at approximately 25 °C during the measurements. The permeability of carbon felt was measured according to a previously reported method [41]. Gas collection measurements were taken on fully charged full cells at a constant charging voltage of 2 V for 20 h.

### Electrochemical Measurements

Electrochemical measurements were taken using a CHI660E electrochemical workstation (Chenhua Instruments, China). Electrochemical impedance spectroscopy (EIS) and cyclic voltammetry (CV) were performed in a conventional three-electrode setup with carbon felt as the working electrode, saturated calomel electrode (SCE) as the reference electrode, and a platinum foil as the counter electrode. EIS was measured in 0.05 M Zn(OH)_4_^2−^ electrolyte without and with various isomalt concentrations, with frequency range from 10^–2^ to 10^5^ Hz at a polarization potential of  − 1.54 V vs. SCE. CV tests were performed between -1.8 and -1.0 V at a scan rate of 10 mV s^−1^. Linear sweep voltammetry (LSV) was further performed in the same electrolyte and electrode configuration, sweeping cathodically from the open-circuit potential to  − 1.8 V vs. SCE at a rate of 2 mV s^−1^. Chronoamperometric (CA) measurements were taken to evaluate transient current responses at  − 1.65 V. Differential capacitance was calculated from cyclic voltammetry recorded at scan rates of 10, 20, 30, 40, and 50 mV s⁻^1^ within non-Faradaic potential window of  − 1.0 ~  − 0.5 V vs. SCE, using a 4-cm^2^ carbon-felt electrode. Full-cell tests of alkaline ZIFB were conducted using a NEWARE battery tester (CT-4008 T, China). The effective electrode area of the flow cell was 4 cm^2^, and 7 mL of electrolyte was used for each side. The positive electrolyte was 0.4 M Na_4_[Fe(CN)_6_] in 3 M KOH, with or without isomalt additive, while the negative electrolyte contained 0.2 M Zn^2+^ in 3 M KOH. A voltage window of 0.8–2.0 V was employed. The polarization curve was measured at current densities from 0 to 400 mA cm^−2^. In addition, self-discharge tests were performed under open-circuit conditions (applied current = 0) to compare the voltage decay behavior of differnet electrolytes. Furthermore, discharge polarization and power density curves of the ZIFB were obtained to assess the internal resistance (ASR) and output characteristics of cells with and without isomalt additives.

### Computational Methods

Density functional theory calculations were conducted using Gaussian 16 software with B3LYP functional and 6-31G(d) basis set to determine the adsorption behavior and electronic interactions of isomalt on carbon-based surfaces. Adsorption energies were calculated to evaluate the binding strength of isomalt on carbon-based surfaces and to assess its influence on interfacial Zn deposition behavior. The CI-NEB method was adopted to determine the energy barrier of Zn migration on varied lattice, which was performed on CP2K 2024 package. Molecular dynamics (MD) simulations were carried out with GROMACS 2020.3 using the OPLS-AA force field to model water and electrolyte species. A time step of 0.5 fs was used, and the system was first equilibrated under NVT ensemble for 20 ps at 298.15 K, followed by a 20-ns production run under NPT conditions (1 atm). The simulation box was set to be 3 × 3 × 3 nm^3^, which contains 500 water molecules, 20 ZnCl_2_ molecules, and 2 isomalt molecules. Temperature and pressure were controlled using Berendsen thermostat and barostat. Finite element simulation of the electrodeposition process on the Zn anode surface was conducted using COMSOL Multiphysics 6.3. An initial zinc surface with an area of 1 × 2 μm^2^ and a roughness of 0.044 μm was generated using Gaussian and uniform random distribution functions. The model employed an ultrafine mesh with a maximum grid size of 0.015 μm. The Butler–Volmer equation, Faraday’s law of electrolysis, and the Nernst–Einstein relationship were adopted to describe and govern the electrochemical reactions at the Zn surface. The dynamic analysis of Zn electrodeposition was achieved using COMSOL’s deformable mesh feature, and the MUMPS solver was used to handle the model-solving process, assuming no parasitic reactions, which corresponds to 100% electrodeposition efficiency.

## Results and Discussion

### Mechanism of Hydrogen-Bonding Regulation and Interfacial Adsorption by Isomalt

**Fig. 2:**
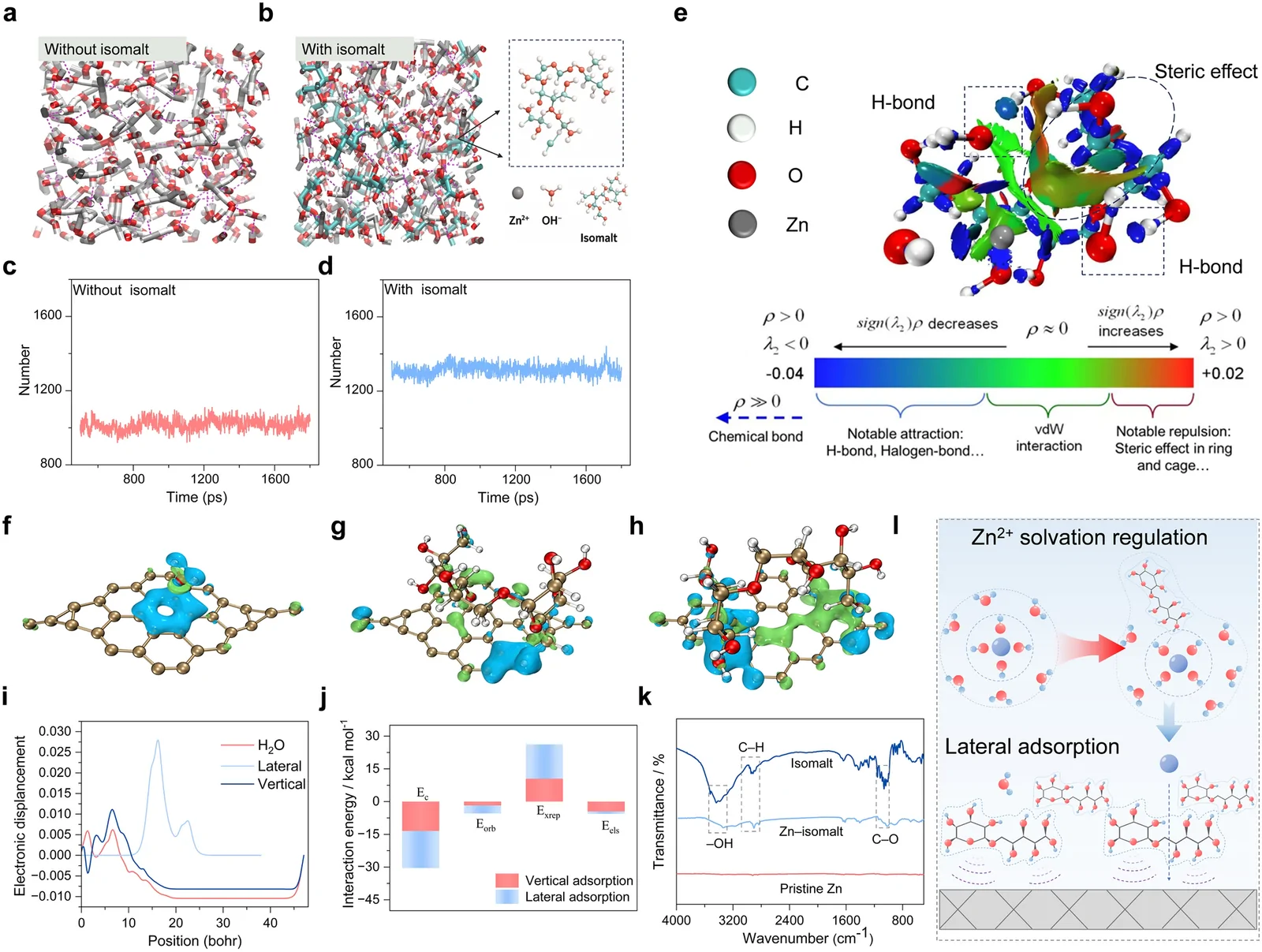
**a-b** Snapshots of pristine electrolytes with/without the isomalt obtained from MD simulations, and **c–d** number of H-bonds in varied electrolyte systems. **e** IRI analysis of isomalt-Zn(OH)_4_^2−^ interactions. Differential charge density of **f** water molecule, **g** lateral and **h** vertical adsorption configurations. **i** Electronic displacement of water molecule, lateral and vertical adsorption configurations. **j** Energy decomposition for the interaction energy between isomalt and graphite (001) surface (E_c_: Coulombic correlation, E_orb_: Orbital, E_xrep_: Exchange-repulsion, E_els_: Electrostatic). **k** FTIR spectra of the zinc foil soaked in a saturated aqueous solution of isomalt for 5 days. **l** Mechanistic explanation of the effect of isomalt on bulk H-bonding network and electrolyte/electrode interface

To evaluate whether isomalt can concurrently regulate the bulk-electrolyte environment and the electrode interface, it was introduced as an additive into the alkaline electrolyte (Fig. S1a). Electrostatic potential (ESP) analysis (Fig. S1b) shows that the negative potential is mainly localized around the oxygen atoms of the hydroxyl and ether groups, indicating that isomalt is well suited to interact with both water molecules and zincate species. We first examined its influence on the bulk H-bond network. Molecular dynamics (MD) simulations show that the total number of H-bonds increases from ~ 1000 to ~ 1300 after isomalt incorporation (Fig. 2a–d), suggesting that the abundant hydroxyl groups of isomalt participate extensively in H-bond donation and acceptance to form a denser network. Interaction region indicator (IRI) analysis further reveals the interaction between isomalt and Zn(OH)_4_^2−^ (Fig. 2e). Strong hydrogen-bonding interactions are observed between the hydroxyl groups of isomalt and the oxygen atoms of Zn(OH)_4_^2−^, whereas steric repulsion from the rigid molecular skeleton provides local spatial constraint. Such combined attractive and steric effects are expected to stabilize the local coordination environment of zincate species.

The evolution of the H-bond network was further probed by Raman spectroscopy (Fig. S2). Deconvolution analysis (Fig. S3) shows that the fraction of strong H-bonds (2800–3000 cm^−1^) decreases from 8.5% in the pristine electrolyte (PE) to 4.9% at 1 wt% isomalt, while the weakly bonded component (3150–3300 cm^−1^) rises to 66.9%, accompanied by a slight decrease in the non-H-bonded fraction (3400–3600 cm^−1^) [42, 43]. These results suggest that moderate isomalt addition weakens overly strong water–water interactions and reconstructs a more flexible yet cohesive H-bond network [44]. Operando Raman spectroscopy (Fig. S4) shows that the 1 wt% electrolyte exhibits enhanced signals and additional shoulder-like features in the 1250–1500 and 1500–1850 cm^−1^ regions under cathodic polarization. These bands are consistent with C–O/C–H related vibrations and interfacial carbonaceous or oxygen-containing species [45, 46], indicating the interfacial participation and preferential accumulation of isomalt during charging. Meanwhile, after isomalt addition, the O–H stretching region (3200–3600 cm^−1^) narrows, showing a more concentrated O–H envelope and a diminished high-wavenumber tail linked to non-H-bonded/free water [47, 48]. FTIR spectra in the O–H stretching and bending regions show no pronounced peak shift with increasing isomalt concentration (Fig. S5), indicating that the original local water environment is not fundamentally disrupted. Instead, isomalt appears to integrate into the existing network through additional weak interactions. Consistently, deconvoluted FTIR spectra (Fig. S6) show that the uncoupled O–H component remains nearly constant at ~ 60%, supporting the formation of a loose but stabilized H-bond network without changing the dominant bonding motif [43, 49]. In parallel, the red shift observed in the UV–VIS spectra at 230–250 nm (Fig. S7) suggests a change in the local electronic environment associated with this network reorganization.

We next investigated how isomalt regulates the electrode/electrolyte interface. Adsorption calculations on graphite (001) show that the lateral configuration is energetically more favorable than the vertical one, with adsorption energies of  − 0.73 and  − 0.41 eV, respectively (Figs. S8 and S9). Electronic density difference analysis further confirms stronger charge redistribution for lateral adsorption (Fig. 2f–h), with a larger electron displacement than that of water or vertically adsorbed isomalt (Fig. 2i). Energy decomposition analysis shows that dispersion interaction makes the largest contribution in both cases and is markedly stronger for the lateral configuration owing to its more extensive interaction with the delocalized π-electrons of graphite (Fig. 2j) [50]. FTIR, XPS, and differential capacitance measurements further support interfacial adsorption of isomalt-derived species on Zn (Figs. 2k and S10, S11). Together, these results indicate that isomalt not only reconstructs the bulk H-bond network, but also preferentially adsorbs at the carbon interface and deposited Zn to establish a hydroxyl-rich interfacial microenvironment (Fig. 2l). This coupled bulk/interface regulation provides the physicochemical basis for homogenized local zincate transport and more controlled Zn deposition in the subsequent electrochemical process [39].

### Electrochemical Behaviors

**Fig. 3:**
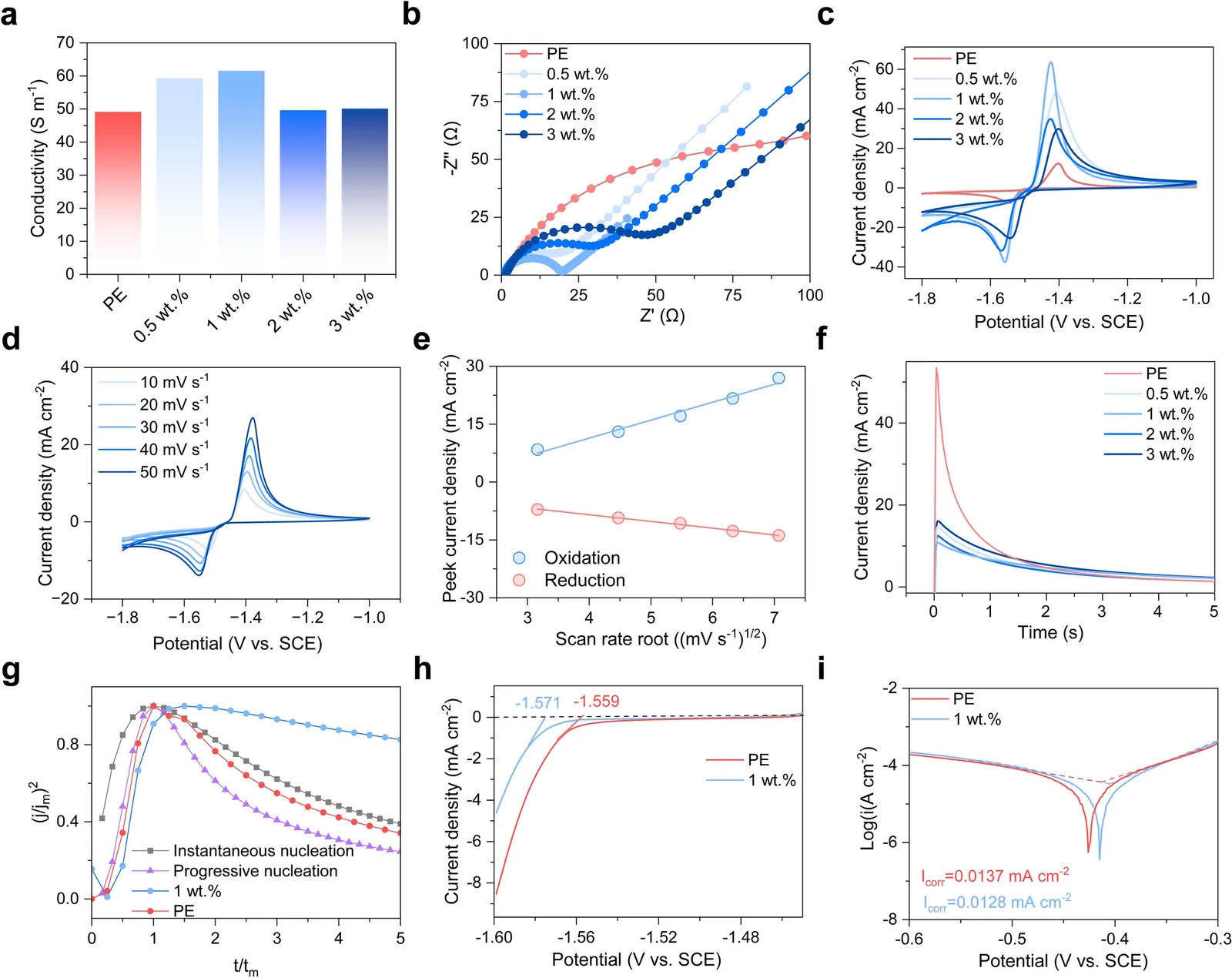
**a** Conductivity, **b** EIS, and **c** CV of PE and PE-isomalt electrolyte with various concentrations. **d–e** CV curves of the 1 wt% electrolyte at different sweep rates and corresponding peak current densities versus square root of scan rates. **f** CA measured in PE and PE-isomalt electrolytes. **g** Comparison of the theoretical dimensionless plots for instantaneous and progressive nucleation process in PE and 1 wt% electrolytes. **h** LSV and **i** Tafel profiles in the PE and 1 wt% electrolytes

Building on the mechanistic picture that isomalt simultaneously reconstructs the bulk H-bond network and establishes a preferentially adsorbed interfacial layer, we next examined whether this coupled regulation translates into improved electrochemical transport and interfacial stability. As shown in Fig. 3a, the ionic conductivity first increases and then decreases with increasing isomalt content, reaching a maximum at 1 wt%, indicating that moderate isomalt incorporation optimizes ion transport, whereas excessive additive loading likely introduces over-association within the electrolyte. Consistent with this trend, electrochemical impedance spectroscopy (EIS) shows that the 1 wt% electrolyte delivers the lowest charge-transfer resistance among all samples (Fig. 3b), demonstrating the most favorable interfacial charge-transfer kinetics. This result suggests that the isomalt-regulated bulk/interface microenvironment is most effective at 1 wt%, where H-bond reorganization and interfacial adsorption are balanced without sacrificing ionic mobility.

The kinetic advantage of the 1 wt% electrolyte is further reflected in the cyclic voltammetry (CV) profiles. Compared with PE, the 1 wt% system exhibits a smaller anodic/cathodic peak separation and higher peak current densities (Fig. 3c), indicating improved reversibility and faster Zn plating/stripping kinetics. Scan-rate-dependent CV measurements show that the redox current increases progressively with scan rate (Fig. 3d), while the peak current displays a good linear relationship with the square root of scan rate (Fig. 3e), consistent with a diffusion-governed process. Moreover, the calculated diffusion coefficients (Table S2) further confirm the enhanced electrochemical activity of the 1 wt% electrolyte. Together, these results indicate that moderate isomalt addition facilitates mass transport and charge exchange under dynamic electrochemical conditions, in agreement with the conductivity and EIS results.

We then evaluated the influence of isomalt on Zn deposition behavior. Chronoamperometric curves show that the 1 wt% electrolyte maintains a smoother and more stable current response during Zn plating than PE (Fig. 3f), suggesting a more uniform deposition process with reduced local current fluctuation. To further probe the nucleation mode, the normalized current–time transients were compared with the Scharifker–Hills model (Fig. 3g). The PE exhibits behavior between instantaneous and progressive nucleation, whereas the 1 wt% electrolyte shows a distinct evolution with a delayed peak time and a more sustained current response. This behavior is consistent with a modified nucleation/growth process in which the interfacial microenvironment created by isomalt promotes more homogeneous three-dimensional deposition rather than localized runaway growth. In parallel, solution viscosity increases with additive content (Fig. S12a), a trend that reflects the progressive formation of bulk H-bond networks. Notably, this viscosity increase does not reduce permeability (Fig. S12b), indicating no additional pumping loss or decrease in overall system efficiency in practice. This can be attributed to the increased electrode porosity (Fig. S12c) resulting from the denser and more uniform Zn deposition achieved with isomalt compared to PE.

Finally, the impact of isomalt on parasitic reactions was assessed. Linear sweep voltammetry reveals that the 1 wt% electrolyte shifts the HER onset toward more negative potential relative to PE (Fig. 3h), indicating suppressed hydrogen evolution. Figure S13 demonstrates that the hydrogen-evolution volume is markedly lower in the isomalt-containing electrolyte than in PE, directly evidencing isomalt’s effectiveness in suppressing hydrogen evolution. Tafel analysis further shows a lower corrosion current density for the 1 wt% electrolyte (Fig. 3i), confirming improved corrosion resistance. Taken together, these results demonstrate that isomalt does not merely act as a conductivity modifier; rather, by coupling bulk H-bond regulation with interfacial microenvironment programming, it improves local transport, moderates Zn nucleation/growth behavior, and suppresses water-driven parasitic reactions. This electrochemical response provides direct functional evidence that the isomalt-conditioned porous electrode is more capable of supporting reversible and stable Zn deposition.

### Surface Evolution of Zn Deposition

The stabilizing effect of isomalt at the electrode/electrolyte interface is expected to favor more uniform and compact Zn deposition. To further clarify how this additive influences the spatial evolution of electrodeposition, finite element simulations were performed to monitor the distributions of local current density and surface height on a simplified Zn substrate during different deposition stages. In the simulation, an initial Zn surface with dimensions of 1 × 2 μm^2^ and an average roughness of 0.044 μm were generated by combining a Gaussian distribution with geometric random functions, so as to approximate the irregular topography of a realistic Zn deposit surface. Based on this initial surface, the evolution of deposition behavior in PE and 1 wt% electrolyte was comparatively analyzed.

**Fig. 4:**
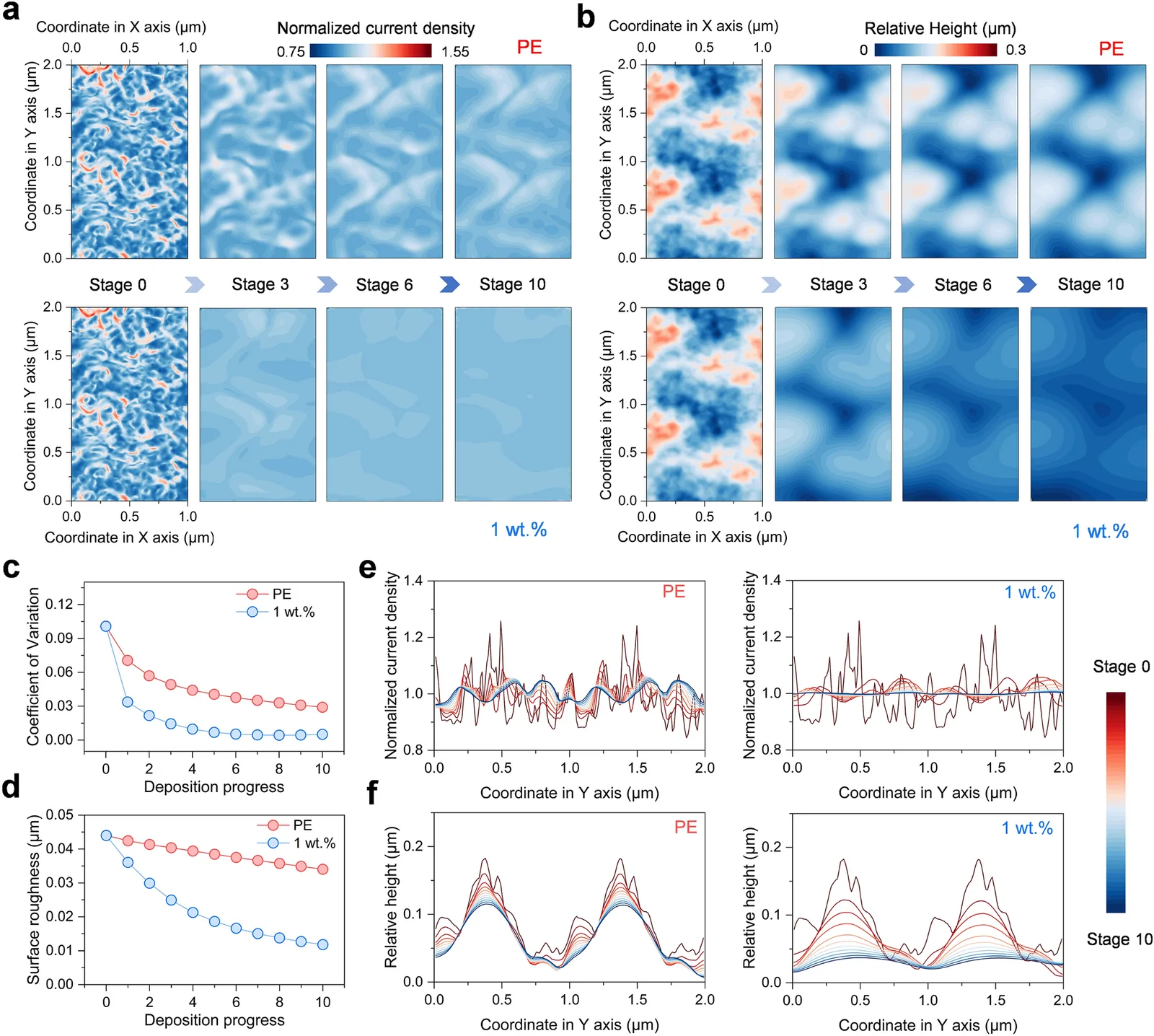
Evolution of **a** normalized current density and **b** relative height on the Zn anode surface with the electrolyte PE and 1 wt%. Change in **c** the uniformity of current density and **d** surface roughness of the Zn anode during the complete electrodeposition process with different electrolytes. **e** Variation of normalized current density and **f** variation of relative height on the Zn anode surface along the Y-axis of X = 0.5 μm in PE and 1 wt% electrolyte

As shown in Fig. 4a, the normalized current density distribution in PE gradually becomes less heterogeneous as deposition proceeds, but a clear spatial gradient remains even at the final stage, indicating persistent local current concentration on surface protrusions. Such nonuniform current distribution implies that certain regions continue to experience preferential Zn growth, which would amplify morphological instability during extended deposition. In contrast, the electrolyte containing 1 wt% isomalt exhibits a much more homogeneous current density distribution throughout the deposition process. With increasing deposition time, the local current density becomes progressively leveled across the entire electrode surface, suggesting that isomalt effectively mitigates localized current focusing and promotes a more evenly distributed electrochemical reaction.

A similar tendency is observed in the evolution of surface height (Fig. 4b). In PE, the relative height distribution remains highly uneven from the initial to the final stage, indicating continuous amplification of surface fluctuations during Zn growth. By comparison, in the 1 wt% electrolyte, the height profile gradually becomes flatter and more uniform as deposition proceeds, demonstrating that the additive suppresses roughness buildup and favors smoother deposition. Quantitative analysis further supports this conclusion. The coefficient of variation of current density decreases much more rapidly in the isomalt-containing electrolyte than in PE (Fig. 4c), while the surface roughness also drops more significantly during deposition (Fig. 4d). To provide a more detailed view, the normalized current density and relative height profiles extracted along the Y-axis at *X* = 0.5 μm (Fig. 4e, f), together with those along the X-axis at *Y* = 1 μm (Fig. S14), consistently show reduced fluctuation amplitude in the presence of isomalt. These simulation results demonstrate that isomalt homogenizes local zincate transport and suppresses surface amplification during Zn growth, thereby providing a theoretical basis for the more uniform and compact Zn deposition observed experimentally.

### Characterization for Zn Deposition

**Fig. 5:**
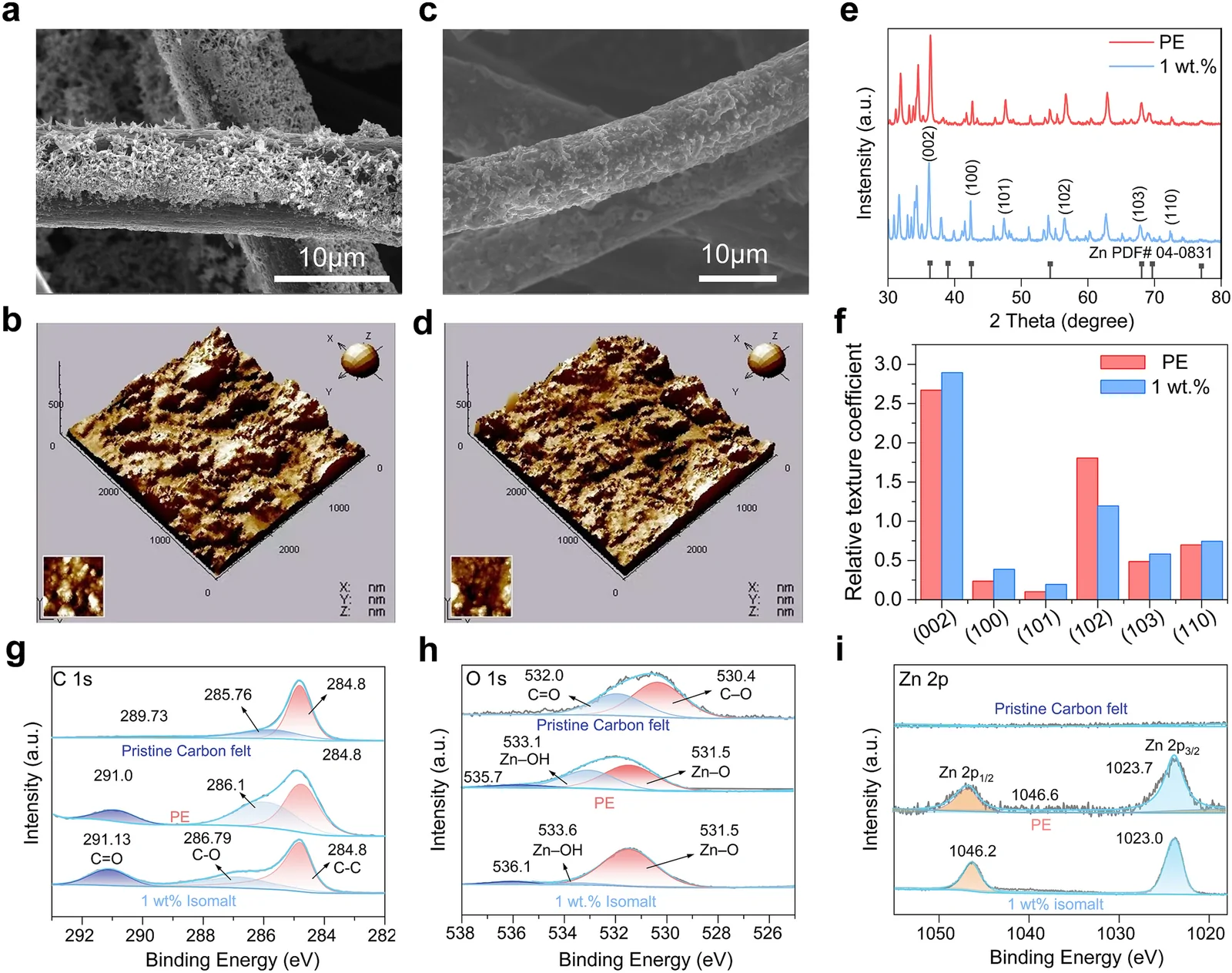
SEM of deposited Zn on carbon felt in **a** PE and **c** 1 wt% electrolyte. AFM images of deposited Zn on Zn foil at 20 mA cm^−2^ in **b** PE and **d** 1 wt% electrolyte. **e** XRD patterns and **f** Calculated relative texture coefficient of different crystal planes of carbon felt cycled in PE and 1 wt% electrolyte. XPS spectra of **g** C 1*s*, **h** O 1*s*, and **i** Zn 2*p* for carbon felt cycling in PE and 1 wt% electrolyte

Electrochemical analysis has shown that 1 wt% isomalt improves Zn plating/stripping reversibility and suppresses parasitic hydrogen evolution. To directly visualize how this interfacial microenvironment regulation affects Zn deposition, we further examined the morphology, crystallographic texture, and surface chemistry of the cycled carbon felt. SEM images show that Zn deposited in PE forms a loose and dendritic morphology on the carbon felt surface (Fig. 5a), indicative of highly nonuniform nucleation and growth. In contrast, the electrode cycled in the 1 wt% electrolyte exhibits a much denser and smoother deposit that covers the carbon substrate more uniformly (Fig. 5c), consistent with a more regulated deposition process. AFM analysis further supports this trend: The surface deposited from (PE) is significantly rougher, whereas the isomalt-containing electrolyte yields a flatter topography with reduced surface roughness (Fig. 5b, d). These results collectively indicate that isomalt suppresses localized Zn accumulation and promotes more homogeneous deposition over the porous carbon framework.

Elemental mapping further reveals the spatial distribution of the deposited species (Figs. S15–S17). Pristine carbon felt is mainly composed of carbon with only trace oxygen, whereas the electrode cycled in PE shows pronounced and nonuniform Zn and O signals, consistent with irregular Zn deposition accompanied by surface side products. By comparison, the electrode cycled in the 1 wt% electrolyte exhibits a more continuous Zn distribution together with a more homogeneous oxygen signal, supporting the formation of a more uniform interfacial environment during deposition. This result is consistent with the proposed role of isomalt in conditioning the porous-electrode surface and homogenizing local transport. The structural consequence of this regulation is reflected in the XRD patterns (Fig. 5e). Both samples display the characteristic reflections of metallic Zn, but their relative intensities differ markedly. In PE, the deposited Zn shows relatively stronger contributions from the non-basal orientations, whereas the isomalt-containing electrolyte gives rise to a more pronounced (002) reflection with weakened (102) intensity. The corresponding relative texture coefficients further quantify this evolution (Fig. 5f). Consistently, calculations of molecular adsorption energies and Zn atom diffusion energies demonstrate that the presence of isomalt enhances the preference for the (002) orientation (Fig. S18). This result suggests that isomalt not only improves deposition uniformity, but also biases Zn growth toward a more favorable crystallographic texture, which is generally associated with more compact growth parallel to the substrate rather than vertically amplified dendritic propagation.

XPS analysis further reveals differences in surface chemistry between the two electrodes (Fig. 5g–i). In the C 1*s* spectra, pristine carbon felt is dominated by C–C and C–O contributions, while the electrode cycled in PE shows stronger oxidized carbon features, indicative of more severe interfacial side reactions (Fig. 5g). By contrast, the isomalt-containing electrode retains a carbon environment enriched with oxygen-containing organic species, consistent with adsorption of isomalt-derived functionalities. In the O 1*s* spectra, the pristine-electrolyte electrode exhibits a more evident Zn–OH contribution in addition to Zn–O (Fig. 5h), suggesting enhanced hydroxide-related byproduct formation. This Zn–OH signal is clearly reduced after cycling in the isomalt-containing electrolyte, consistent with suppressed water-driven parasitic reactions and a more stable Zn-containing interphase. The Zn 2*p* spectra also show stronger and better-defined Zn signals for the isomalt-modified electrode (Fig. 5i), in line with the formation of a more continuous and better regulated Zn deposit. Taken together, these morphological, structural, and spectroscopic results confirm that isomalt-induced bulk/interface regulation effectively converts the porous carbon host into a more deposition-directing electrode environment, thereby enabling compact Zn growth with reduced side reactions.

### Performance Evaluation of Full Cell

**Fig. 6:**
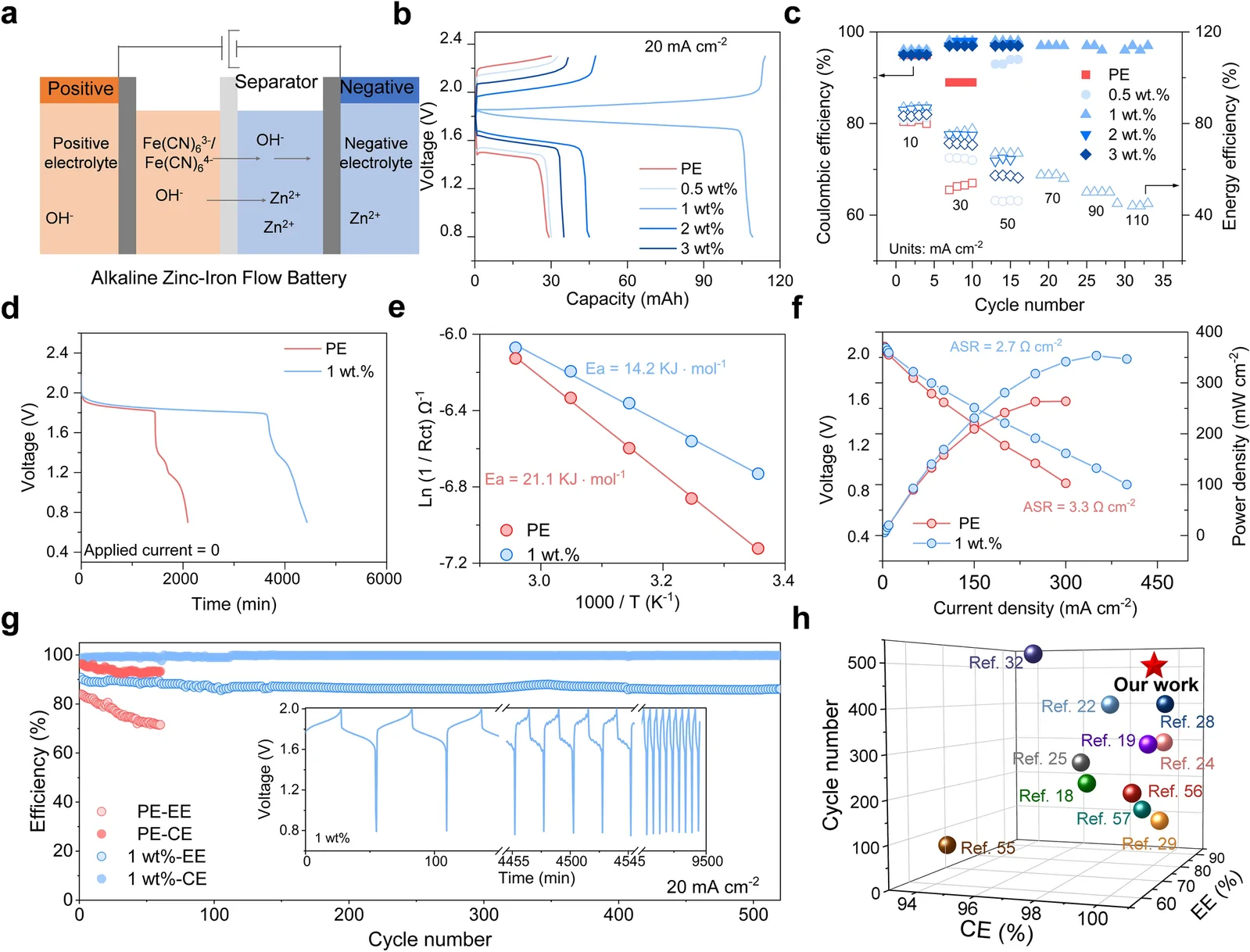
**a** Schematic illustration for the mechanism of the alkaline ZIFB. **b** Capacity profiles at various isomalt concentrations (0–3 wt%) at 20 mA cm^−2^_._ **c** Rate performance at current density ranging from 10 to 110 mA cm^−2^. **d** Self-discharge curves of the full cell with PE and 1 wt% electrolyte. **e** Activation energy in PE and 1 wt% electrolyte. **f** Polarization and power density curves of the cell with PE and 1 wt% electrolyte. **g** Comparison of long-term galvanostatic charging/discharging of the alkaline ZIFBs with and without isomalt at 20 mA cm^−2^. **h** Performance comparison with the reported literature

Given that isomalt suppresses parasitic hydrogen evolution and regulates Zn deposition through coupled bulk/interface modulation, we next evaluated whether these interfacial benefits can be translated into improved full-cell performance in ZIFBs (Figs. 6a and S19). Na_4_[Fe(CN)_6_] in KOH is a commonly employed catholyte for alkaline ZIFBs [51–54]. It is found that Na_4_[Fe(CN)_6_] maintains good electrochemical reversibility and chemical stability in 3 M KOH under testing conditions according to CVs (Fig. S20) for the fresh positive electrolyte, the positive electrolyte after standing for 5 days, and the positive electrolyte after 200 charging–discharging cycles. Besides, electrochemical analysis was also conducted on the positive electrolyte with and without isomalt. CV, EIS, and LSV (Fig. S21a–c) results demonstrate that even if isomalt migrates through the diaphragm, it causes negligible influence on the electrochemical performance of the positive electrode reaction. As shown in Fig. 6b, the cell using 1 wt% electrolyte exhibits markedly lower polarization and a higher discharge capacity than that using PE, indicating substantially improved reaction reversibility under practical flow-battery operation. This advantage becomes more evident in the rate-dependent efficiency analysis (Fig. 6c). The 1 wt% electrolyte maintains a stable Coulombic efficiency above 98.2% over a wide current–density range, while the energy efficiency gradually decreases from 87.5% to 45.0% as the current density increases from 10 to 110 mA cm^−2^. In contrast, the battery using PE undergoes much more rapid efficiency deterioration, with both coulombic and energy efficiencies declining sharply even within a narrower current–density range. These results indicate that isomalt enables more robust Zn plating/stripping reversibility and lower polarization loss during dynamic operation. The beneficial effect of isomalt is further reflected in the self-discharge behavior. As shown in Fig. 6d, the open-circuit voltage of the battery with PE decays to ~ 0.5 V after 2000 min, whereas the isomalt-containing system still maintains a voltage of ~ 1.3–1.4 V even after 4000 min. Isomalt reconstructs the H-bond network and lowers the activity of free water, thereby suppressing hydrogen evolution. Meanwhile, its preferential interfacial adsorption reduces dendrite-induced local corrosion and side reactions. Collectively, these effects slow spontaneous charge loss under open-circuit conditions, leading to substantially lower self-discharge. Such behavior is fully consistent with the earlier evidence for reduced hydrogen evolution reaction (HER), lower corrosion tendency, and more regulated Zn deposition. To further clarify the kinetic origin of the improved cell performance, the activation energy and polarization characteristics were analyzed. The apparent activation energy decreases significantly from 21.1 kJ mol^−1^ for PE to 14.2 kJ mol^−1^ for the 1 wt% electrolyte (Fig. 6e), indicating that the isomalt-regulated electrolyte/electrode microenvironment lowers the interfacial kinetic barrier. Consistently, the polarization profiles show that the area-specific resistance decreases from 3.3 to 2.7 Ω cm^2^ after isomalt addition (Fig. 6f), confirming reduced cell resistance and facilitated charge-transfer processes. Consequently, the corresponding power-density curve shifts toward higher current density, and the maximum power density increases from 250 to 350 mW cm^−2^. These results demonstrate that isomalt improves not only interfacial stability, but also the overall reaction kinetics of the operating flow battery. The long-term cycling data provide the most direct validation of this design strategy. As summarized in Figs. 6g, h, S22 and Table S3 [55–57], the optimized ZIFB delivers a well-balanced combination of high coulombic efficiency (~ 98%), high energy efficiency (~ 87%), outstanding capacity retention (with a low capacity decay rate of only 0.009% per cycle) and stable cycling over 500 cycles, surpassing the performance of many previously reported alkaline ZIFBs. Besides, UV–VIS (Fig. S23a) and Ramon spectra (Fig. S23b) indicate negligible isomalt migration to the positive side and no evident changes in concentration or molecular structure of isomalt in the negative side after 500 cycles. Moreover, we compared isomalt with a reported polyhydroxyl-group-containing additive (i.e., sorbitol) [58]. The SEM images and cycling tests (Fig. S24) indicate that isomalt provides a greater enhancement of Zn deposition morphology and long-term stability. Taken together, these full-cell results confirm that isomalt does not merely function as a molecular additive in the electrolyte; rather, by conditioning both the bulk H-bond network and the porous-electrode interfacial microenvironment, it enables more reversible Zn redox chemistry and more durable operation under realistic flow-battery conditions.

## Conclusion

In summary, this work shows that isomalt functions beyond a conventional electrolyte additive in alkaline ZIFBs and instead acts as a polyhydroxy molecular regulator that couples bulk–electrolyte reconstruction with interfacial microenvironment control. In the bulk phase, isomalt reorganizes the local H-bond network, moderates water activity, and thereby suppresses parasitic hydrogen evolution. At the porous-electrode interface, its preferential lateral adsorption establishes a hydroxyl-rich interphase that regulates local zincate transport and biases Zn deposition toward a more favorable growth mode. Through this coupled regulation, the optimized 1 wt% electrolyte lowers the charge-transfer resistance to 18.4 Ω and reduces the apparent activation energy to 14.2 kJ mol^−1^, leading to improved Zn plating/stripping reversibility, reduced polarization, and accelerated cell-level kinetics. As a result, the corresponding ZIFB delivers high Coulombic and energy efficiencies together with stable cycling over 500 cycles. More broadly, these findings suggest that polyhydroxy molecules can serve not only as electrolyte modifiers, but also as interfacial programming units for porous electrodes. This work therefore establishes a molecularly enabled bulk/interface coupling strategy for stabilizing Zn anodes in aqueous flow-battery systems and provides a broader design framework for directing metal deposition in multivalent energy-storage chemistries.

## Supplementary Information

Below is the link to the electronic supplementary material.

Supplementary file1 (DOCX 25514 kb)
