# Supplementary material for: Polyhydroxy Molecular Regulator Enabled Interfacial Microenvironment Programming for Alkaline Zinc–Iron Flow Batteries
Source: Nanomicro Lett. 2026 Sep 14;19:47. doi: 10.1007/s40820-026-02333-2 (PMC13572358; doi:10.1007/s40820-026-02333-2)
Supplement: Supplementary file 1 [file 40820_2026_2333_MOESM1_ESM.docx]

Supporting Information for

**Polyhydroxy Molecular Regulator Enabled Interfacial Microenvironment Programming for Alkaline Zinc-Iron Flow Batteries**

Hui Chen^1#^, Lukang Han^3#^, Shaohua Han^2^, Weijie Fan^2^, Xuhong Yin^1^, Junqing Pan^3^, Manal S. Ebaid^4^, Yuxi Song^1,^ *, Fuyu Chen^1,^ *, and Jiang Zhou^2,^ *

^1^ School of Materials Science and Engineering, Yancheng Institute of Technology, Yancheng 224051, P. R. China

^2^ School of Materials Science and Engineering, Hunan Provincial Key Laboratory of Electronic Packaging and Advanced Functional Materials, Central South University, Changsha 410083, P. R. China

^3^ State Key Laboratory of Chemical Resource Engineering, College of Chemistry, Beijing University of Chemical Technology, Beijing 100029, P. R. China

^4^ Department of Chemistry, College of Science, Northern Border university, Arar 73213, Saudi Arabia

#Hui Chen and Lukang Han contributed equally to this work.

*Corresponding authors. E-mail: [yxsong@ycit.edu.cn](mailto:yxsong@ycit.edu.cn) (Yuxi Song); fychen@ycit.cn (Fuyu Chen); [zhou_jiang@csu.edu.cn](mailto:zhou_jiang@csu.edu.cn) (Jiang Zhou)

**S1 Supplementary Text**

***S1.1 Materials***

All reagents used in this study were of analytical grade and employed without further purification. Isomalt (purity 99%) was purchased from Macklin Biochemical Co., Ltd. (Shanghai, China). Zinc oxide (ZnO), potassium hydroxide (KOH), and sodium ferrocyanide (Na_4_[Fe(CN)_6_]) were obtained from commercial suppliers and used as received. Carbon felt (CF) was employed as the electrode substrate after pretreatment by acid washing and drying to remove surface impurities and enhance electrochemical activity. While the zinc foil was rinsed with 0.5 M HCl (Xinyue Chemical Reagent, China) to remove the contaminants, and then cleaned up with deionized water.

***S1.2 Electrolyte Composition***

The alkaline anolyte was prepared by dissolving 0.2 M ZnO and 3.4 M KOH in deionized water under constant stirring at 40 °C until a clear solution was obtained. Isomalt was introduced into the base electrolyte at varying concentrations (0, 0.5, 1, 2, and 3 wt.%) to investigate its effect as a functional additive. The catholyte was composed of 0.4 M Na_4_[Fe(CN)_6_] and 3 M KOH. All electrolytes were filtered to remove insoluble impurities and stored under a nitrogen atmosphere to minimize oxidative degradation and ensure chemical stability before use.

***S1.3 Material characterizations***

The morphology was analyzed using Scanning Electron Microscopy (SEM, FEI Nova Nano SEM 450), in conjunction with Energy-Dispersive X-ray Spectroscopy (EDS, Oxford AZtec X-MaxN 80). X-ray Diffraction (XRD, PANalytical X’Pert^3^ Powder, Cu Kα, λ = 1.5406 Å) radiation was conducted ranging from 30° to 90° The relative texture coefficients (RTCs) of different Zn lattice planes for varied concentrations of isomalt were calculated according to the following formula:

$\text{RTC=}\frac{\text{I}_{\text{(hkl)}}\text{/}\text{I}_{\text{0(hkl)}}}{\frac{\text{1}}{\text{N}}\sum_{i=1}^{N} (\text{I}_{\text{(hkl)}}\text{/}\text{I}_{\text{0}\left( \text{hkl} \right)}\text{)}}$	(S1)

where I_(hkl)_ is the intensity obtained from the textured sample, N is the number of crystals involved in the calculation, and I_0(hkl)_ is the intensity of the standard-oriented sample. X-ray Photoelectron Spectroscopy (XPS, Thermo ESCALAB 250Xi, 10 kV) was used to probe the surface chemical composition and the oxidation states of the elements present on the zinc-deposited CF electrode. Besides, Atomic Force Microscopy (AFM, Picoplus 2500, USA) was utilized to characterize the deposited surface. Fourier Transform Infrared Spectroscopy (FTIR, Thermo Nicolet NEXUS-670) was conducted to investigate the chemical interactions. The Raman spectra (Horiba France SAS, LabRAM Odyssey) were collected using a 532 nm excitation laser with a laser power of 10 mW. Ultraviolet–visible spectroscopy (UV-Vis, Shimadzu UV-2450) was employed to assess the optical absorbance behavior of electrolytes with varying isomalt concentrations. The viscosity of electrolyte was tested using (DV2T, AMETEK Brookfield, USA). The porosity of electrodes was investigated by mercury intrusion porosimetry (MIP) using a Micromeritics AutoPore V 9600 apparatus (version 2.03.00, Micromeritics Instrument Corporation, USA). The analysis was performed in a pressure range from 0.10 psia to 61,000.00 psia. The contact angle of mercury was set to 130° and the mercury temperature was maintained at approximately 25 °C during the measurements. The permeability of carbon felt was measured according to a previously reported method [*doi.org/10.1016/j.cej.2025.166815*]. Gas collection measurements were performed on fully charged full cells at a constant charging voltage of 2 V for 20 h.

***S1.4 Electrochemical Measurements***

Electrochemical measurements were conducted using a CHI660E electrochemical workstation (Chenhua Instruments, China). Electrochemical impedance spectroscopy (EIS) and cyclic voltammetry (CV) were performed in a conventional three-electrode setup with carbon felt as the working electrode, saturated calomel electrode as the reference electrode, and a platinum foil as the counter electrode. EIS was carried out in 0.05 M Zn(OH)_4_^2−^ electrolyte without and with various isomalt concentrations, with frequency range from 10^-2^ to 10^5^ Hz at a polarization potential of -1.54V vs. SCE. CV tests were performed between -1.8 V and -1.0 V at a scan rate of 10 mV s^-1^. The diffusion coefficient *D* was calculated according to the Randles-Sevcik equation, as follows:

$\text{i}_{\text{p}}\text{=2.69×}\text{10}^{\text{5}}\text{n}^{\text{3/2}}\text{AD}^{\text{1/2}}\text{C}\text{v}^{\text{1/2}}\text{ }\text{ }$(S2)

In this equation, $i_{p}$is the peak current in the cyclic voltammogram; $n$is the number of electrons transferred in the redox process; $D$is the diffusion coefficient of the active species; $C$is the concentration of the active species in the test solution; $\nu$is the scan rate used in the measurement. Linear sweep voltammetry (LSV) was further performed in the same electrolyte and electrode configuration, sweeping cathodically from the open-circuit potential to -1.8 V vs. SCE at a rate of 2 mV s^-1^. The activation energy (E_a_) can be determined using the Arrhenius relationship:

$\text{1}/{\text{R}_{\text{ct}}}\text{=Aexp}\left( {\text{-E}_{\text{a}}}/\text{RT} \right)$	(S3)

where R_ct_, A, R, and T represent the charge-transfer resistance, the frequency factor, the gas constant, and the absolute temperature, respectively. Chronoamperometric (CA) measurements were conducted to evaluate transient current responses at -1.65V. Instantaneous nucleation and progressive nucleation are expressed as follows:

Instantaneous：

$\left( \frac{j}{j_{m}} \right)^{2}=1.9542\left( \frac{t}{t_{m}} \right)^{-1}\left\{ 1-exp\left[ -1.2564\left( \frac{t}{t_{m}} \right) \right] \right\}^{2}$(S4)

Progressive：

$\left( \frac{j}{j_{m}} \right)^{2}=1.2254\left( \frac{t}{t_{m}} \right)^{-1}\left\{ 1-exp\left[ -2.3367\left( \frac{t}{t_{m}} \right)^{2} \right] \right\}^{2}$(S5)

The nucleation pattern depends on comparing dimensionless form (*j/j_m_*)^2^ vs. (*t/t_m_*) current-time transient charts with models originated from 3D instantaneous and progressive nucleation.

Differential capacitance was calculated from cyclic voltammetry recorded at scan rates of 10, 20, 30, 40, and 50 mV s⁻¹ within non-Faradaic potential window of -1.0~-0.5 V vs. SCE, using a 4 cm^2^ carbon felt electrode. The full-cell tests of alkaline Zn-Fe flow batteries were conducted using a NEWARE battery tester (CT-4008T, China) to evaluate charge-discharge performance and cycling stability. The effective electrode area of the flow cell was 4 cm^2^, and 7 mL of electrolyte was used for each side. The positive electrolyte was composed of 0.4 M Na_4_[Fe(CN)_6_] in 3 M KOH, with or without isomalt additive, while the negative electrolyte contained 0.2 M Zn^2+^ in 3 M KOH. A voltage window of 0.8-2.0 V was employed. The current density was varied from 10 to 110 mA cm^-2^ to assess rate capability under different isomalt concentrations. Long-term cycling tests were performed at a fixed current density to monitor its durability. The polarization curve was measured at current densities from 0 to 400 mA cm^−2^. In addition, self-discharge tests were performed under open-circuit conditions (applied current = 0) to compare the voltage decay behavior of the PE and 1 wt.% isomalt electrolyte. Furthermore, discharge polarization and power density curves of the Zn-Fe flow cell were obtained to assess the internal resistance (ASR) and output characteristics of cells with and without isomalt additives.

***S1.5 Computational Methods***

To gain molecular-level insights into the role of isomalt at the electrode interface, density functional theory (DFT) and molecular dynamics (MD) simulations were performed. DFT calculations were conducted using Gaussian 16 software with B3LYP functional and 6-31G(d) basis set to determine the adsorption behavior and electronic interactions of isomalt on carbon-based surfaces. Adsorption energies were calculated to evaluate the binding strength of isomalt on carbon-based surfaces and to assess its influence on interfacial Zn deposition behavior. The CI-NEB method was adopted to determine the energy barrier of Zn migration on varied lattice, which was performed on CP2K 2024 package. MD simulations were carried out with GROMACS 2020.3 using the OPLS-AA force field to model water and electrolyte species. A time step of 0.5 fs was used, and the system was first equilibrated under NVT ensemble for 20 ps at 298.15 K, followed by a 20 ns production run under NPT conditions (1 atm). The simulation box was set to be 3×3×3 nm^3^, which contains 500 water molecules, 20 ZnCl_2_ molecules, and 2 isomalt molecules. Temperature and pressure were controlled using Berendsen thermostat and barostat. The simulations provided atomistic evidence for H-bond-network reorganization and additive-induced interfacial structuring in the alkaline electrolyte.

Finite element simulation of the electrodeposition process on the Zn anode surface was conducted using COMSOL Multiphysics 6.3. An initial zinc surface with an area of 1 μm × 2 μm and a roughness of 0.044 μm was generated using Gaussian and uniform random distribution functions. The model employed an ultrafine mesh with a maximum grid size of 0.015 μm. The Butler-Volmer equation, Faraday’s law of electrolysis, and the Nernst-Einstein relationship were adopted to describe and govern the electrochemical reactions at the Zn surface. The dynamic analysis of Zn electrodeposition was achieved using COMSOL’s deformable mesh feature, and the MUMPS solver was used to handle the model-solving process, assuming no parasitic reactions, which corresponds to 100% electrodeposition efficiency. In this article, the normalized current density data were obtained by dividing the current density at each point on the Zn anode surface by the average current density over the entire surface. The relative height data were derived by subtracting the minimum surface height from the height value at each point.

**S2 Supplementary Figures**

**Fig. S1 a** The structural formula of isomalt. **b** Electrostatic potential (ESP) mapping of a free isomalt molecule.

**Fig. S2** Raman spectra of electrolytes with PE and various concentrations of isomalt.

**Fig. S3** Raman peak fitting analysis of electrolytes with **a** PE. **b** 0.5 wt.%. **c** 1 wt.%. **d** 2 wt.%. **e** 3 wt.% in the region of O─H stretching vibration. **f** The proportions of strong, weak, and non-H-bonds obtained by the integral area of separated peaks in the electrolytes.

**Fig. S4** Operando Raman spectra of **a** PE and **b** 1 wt.% Isomalt.

**Fig. S5** FTIR spectra recorded for isomalt, PE, and PE-isomalt electrolytes.

**Fig. S6** FTIR peak fitting analysis of electrolytes with different Isomalt concentrations. **a** PE. **b** 0.5 wt.%. **c** 1 wt.%. **d** 2 wt.%. **e** 3 wt.%. **f** FTIR peak deconvoluting results with different isomalt concentrations.

**Fig. S7** The UV/Vis spectra of electrolytes with different electrolytes.

**Fig. S8** Configurations of **a** water molecule, **b** Lateral and **c** Vertical adsorption.

**Fig. S9** Adsorption energy of H_2_O, Lateral and Vertical adsorption (with “laterally oriented” referring to the isomalt molecules lying flat on the surface and “vertically oriented” indicating the molecules standing upright.

**Fig. S10** **a** The full XPS spectrum of pure zinc and zinc soaked in saturated Isomalt for 5 days. **b-d** The corresponding high-resolution O 1s, C 1s and Zn 2p spectra.

**Fig. S11** Differential capacitance evolution of PE and isomalt-containing electrolytes

The adsorption of isomalt at carbon felt/electrolyte interface could potentially influence the degree of interfacial charge accumulation, thus determine the value of differential capacitance. In isomalt-containing electrolytes, the differential capacitance is markedly higher than in PE, indicating that isomalt participates in potential-dependent adsorption and reorganization at the electrode/electrolyte interface. Furthermore, after re-immersion in PE, the curve only partially recovers, indicating the dynamic adsorption of isomalt and the stability of the interfacial region.

**Fig. S12** **a** Viscosity of different electrolyte samples, **b** permeability and **c** Porosity of negative electrode during charging

**Fig. S13** Comparison of hydrogen evolution volume between PE and 1 wt.% electrolyte.

**Fig. S14 a** variation of normalized current density and **b** Variation of relative height on the Zn anode surface along the X-axis of Y = 1 μm in PE and 1 wt.% electrolyte.

**Fig. S15** **a** Elemental analysis and **b-c** EDS mapping on the pristine carbon felt.

**Fig. S16** **a** Elemental analysis and **b-d** EDS mapping of zinc deposition on the carbon felt after cycling in PE.

**Fig. S17 a** Elemental analysis and **b-d** EDS mapping of zinc deposition on the carbon felt after cycling (with 1 wt.% isomalt).

**Fig. S18** Comparison of adsorption energy and Zn atom diffusion energy barrier on different Zn crystal facets in PE and 1 wt.% electrolytes.

**Fig. S19** Scheme of Zn-Fe RFB configuration.

**Fig. S20** Stability evaluation of Na_4_[Fe(CN)_6_] in 3 M KOH electrolyte

**Fig. S21** Electrochemical Performance of positive electrolyte with and without 1 wt.% isomalt **a** CV, **b** EIS and **c** LSV

**Fig.S22** Capacity evolution of the alkaline Zn-Fe RFBs with and without isomalt at 20 mA cm^−2^.

**Fig. S23** **a** UV-vis spectra of positive electrolyte and **b** Raman spectra of negative electrolyte before and after cycling.

**Fig. S24** SEM images of electrodes after cycling in **a** PE, **b** sorbitol-containing electrolyte, and **c** isomalt-containing electrolyte, respectively. Corresponding cycling stability comparison: **d** efficiency and **e** capacity.

**S3 Supporting Tables**

**Table S1** Comparison of electrolyte regulation and molecular structures for representative hydroxyl-rich additives relative to isomalt

| Additive | Molecular feature | Main reported function | Limitation compared with isomalt | Ref. |
| --- | --- | --- | --- | --- |
| Glycerol | Small flexible triol | Hydrogen-bond regulation, water activity suppression | Limited hydroxyl number and weak interfacial coverage | [S1] |
| Glucose | Monosaccharide with multiple hydroxyl groups | Solvation/interface regulation | Reducing sugar; possible chemical instability under harsh alkaline/electrochemical conditions | [S2] |
| Xylitol | Linear pentol | Zn²⁺ migration regulation, Zn surface adsorption | Flexible chain; relatively small interfacial footprint | [S3] |
| Sorbitol | Linear hexol | Solvation regulation and Zn interface protection | Flexible molecule; less sterically constrained adsorption layer | [S4] |
| Tartrate | Polyhydroxycarboxylate | Zincate coordination; deposition homogenization | Small hydroxycarboxylate ligand; limited steric shielding; lacks disaccharide-polyol backbone | [S5] |
| THEED | Polyhydroxyamine/chelating agent | Zincate chelation; hydrogen-bond reconstruction; water-migration mitigation | Polyhydroxy amine rather than sugar alcohol; chelation-dominated; lacks rigid sugar ring and large interfacial coverage | [S6] |

**Table S2** Diffusion coefficients for electrolytes with different isomalt contents

| electrolyte | Diffusion coefficients for oxidation | Diffusion coefficients for reduction |
| --- | --- | --- |
| PE | 3.35E-4 | 1.74E-4 |
| 0.5 wt.% | 3.766E-4 | 2.015E-4 |
| 1 wt.% | 4.19E-4 | 2.3E-4 |
| 2 wt.% | 4.09E-4 | 2.25E-4 |
| 3 wt.% | 3.96E-4 | 2.17E-4 |

**Table S3** Performance comparison with the reported literature

| Electrolyte Concentration | Current Density (mA cm⁻²) | Areal Capacity (mAh cm⁻²) | CE | EE | Cycle Stability | Refs. in manuscript |
| --- | --- | --- | --- | --- | --- | --- |
| Neg: 0.4 M Zn(OH)_4_^2-^ + 3.8 M OH⁻;Pos: 0.8 M Fe(CN)_6_^4-^ + 3 M OH⁻ | 200 | 33.6 | 97.41% | 80.68% | ~200 cycles | [18] |
| Neg: 0.3 M ZnO + 3.6M OH^-^;Pos: 0.6 M Fe(CN)_6_^4-^ + 3 M OH⁻ | 80 | ---- | 99.6 | 82.8 | 300 cycles | [19] |
| Neg: 0.5 M ZnCl_2_ + 0.05 M NAM + 3 M KCl; Pos: 0.5 M K_4_Fe(CN)_6_ + 1 M KCl | 20 | 3.57 | ~98 | 84.5 | 400 cycles | [22] |
| Neg(Pos):0.6 mol L⁻¹ [Fe(CN)_6_]^4-^ + 0.3 mol L^-1^  ZnO + 3.6 mol L^-1^ OH⁻ | 40 | 60 | ~99.85% | 88% | 300 cycles | [24] |
| Neg: 0.3 M ZnBr_2_ + 0.3 M EDTA+0.025M DSS; Pos: 0.5 M Fe(CN)_6_^4-^ + 3 M OH⁻ | 40 | ---- | 97% | 82.64% | 250 cycles | [25] |
| Neg: 0.4 mol L⁻¹ ZnAc_2_ + 0.4 mol L^-1^ Na_3_Cit Pos: 0.4 mol L^-1^ Na_4_Fe(CN)_6_ + 0.4 mol L^-1^ K_4_Fe(CN)_6_ + 0.4 mol L^-1^ KCl + 0.4 mol L⁻¹ NaCl | 40 | ---- | 99.8 | 89.5 | 400 cycles | [28] |
| Neg: 0.2 M ZnSO_4_ + 4 M LiBr;  Pos: 0.1 M K_4_Fe(CN)_6_ + LiMn_0.2_Fe_0.8_PO_4_ | 10 | ~16.6 | 99.9% | 84.9% | ~100 cycles | [29] |
| Neg: 0.1 M Zn(OH)_4_^2-^ + 1 mM DIPSO;  Pos: 0.2 M K_4_Fe(CN)_6_ + 1 M NaOH | 80 | 6.67 | 95.6% | 77.9% | 530 cycles | [32] |
| Neg: 1 M ZnCl₂ ; Pos: 0.5 M FeCl₂ + 0.5 M FeCl₃ + 2 M NH₄Cl | 25 | 19.4 | 92% | 78% | 30 cycles | [55] |
| Neg: 0.4 M ZnO + 5.6 M NaOH + 0.2 M Ser; Pos: 0.4 M K₄Fe(CN)_6_ + 4 M NaOH | 50 | 30 | 99.21% | 79.89% | >180 cycles | [56] |
| Neg: 0.4 M Na_2_Zn(OH)_4_ + 0.5 M Na_2_SO_4_;  Pos: 0.8 M Na_4_Fe(CN)_6_ + 3 M KOH | 80 | 18.75 | ~99% | ~88% | 120 cycles | [57] |
| Neg :0.2M Zn(OH)_4_^2-^  +4M NaOH; Pos: 0.4M Na_2_Fe(CN)_4_ | 20 | ~25 | ~99% | 87.5% | 500 cycles | our work |

**Supplementary References**

[S1] X. Wei, J. Guan, Y. Mu, Y. Zou, X. Wei et al., Decoding hydrogen-bond network of electrolyte for cryogenic durable aqueous zinc-ion batteries. Nano-Micro Lett. **18**(1), 127 (2026). <https://doi.org/10.1007/s40820-025-01970-3>

[S2] P. Sun, L. Ma, W. Zhou, M. Qiu, Z. Wang et al., Simultaneous regulation on solvation shell and electrode interface for dendrite-free zn ion batteries achieved by a low-cost glucose additive. Angew. Chem. Int. Ed. **60**(33), 18247-18255 (2021). <https://doi.org/10.1002/anie.202105756>

[S3] S. Li, Y. Xu, X. Xu, L. Shi, L. Sun et al., Improved stability of zinc anodes by a trace amount of xylitol. J. Electron. Mater. **53**(1), 408-417 (2024). <https://doi.org/10.1007/s11664-023-10793-0>

[S4] Y. Quan, M. Yang, M. Chen, W. Zhou, X. Han et al., Electrolyte additive of sorbitol rendering aqueous zinc-ion batteries with dendrite-free behavior and good anti-freezing ability. Chem. Eng. J. **458**, 141392 (2023). <https://doi.org/10.1016/j.cej.2023.141392>

[S5] H. Hu, X. Cheng, J. Wang, T. Xuan, L. Wang, High-performance alkaline zinc flow batteries enabled by functional electrolyte additive containing nucleophilic groups. Electrochim. Acta **531**, 146434 (2025). <https://doi.org/10.1016/j.electacta.2025.146434>

[S6] L. Zhi, T. Li, X. Liu, Z. Yuan, X. Li, Functional complexed zincate ions enable dendrite-free long cycle alkaline zinc-based flow batteries. Nano Energy **102**, 107697 (2022). <https://doi.org/10.1016/j.nanoen.2022.107697>
